# Epidemiological characteristics of pulmonary tuberculosis among children in Shandong, China, 2005–2017

**DOI:** 10.1186/s12879-019-4060-x

**Published:** 2019-05-10

**Authors:** Ning-ning Tao, Yi-fan Li, Yun-xia Liu, Jin-yue Liu, Wan-mei Song, Yao Liu, Hong Geng, Shan-shan Wang, Huai-chen Li

**Affiliations:** 1 Department of Respiratory Medicine, Shandong Provincial Hospital Affiliated to Shandong University, Jinan, China; 2 Department of Biostatistics, School of Public Health, Shandong University, Jinan, China; 3 School of Medicine and Life Sciences, University of Jinan-Shandong Academy of Medical Sciences, Jinan, China; 4 Centers for Tuberculosis Control in Shandong province, Jinan, China

**Keywords:** Pulmonary tuberculosis, Pediatric, Incidence, Mortality

## Abstract

**Background:**

Diagnosis of tuberculosis (TB) in children is challenging. Epidemiological data of childhood pulmonary tuberculosis (PTB) are urgently needed.

**Methods:**

We described trends in epidemiology, clinical characteristics, and treatment outcomes in seven cities of Shandong province, China, during 2005–2017. Data were collected from the China Information System for Disease Control and Prevention.

**Results:**

Among 6283 (2.4% of all PTB) PTB cases aged < 18 years, 56.5% were male patients, 39.3% were smear-positive and 98.6% were new cases. The overall incidence of childhood PTB declined (7.62 to 3.74 per 100,000) during 2005–2017, with a non-significant change of annual percentage after 2010. While the incidence of smear-positive PTB (6.09 to 0.38 per 100,000 population) decreased significantly, but the incidence of smear-negative PTB (1.52 to 3.36 per 100,000 population) increased significantly during 2005–2017. The overall treatment success occurred among 94.2% childhood PTB. Ten children (0.2%) died.

**Conclusion:**

The overall incidence of childhood PTB declined significantly with the disease burden shifting from smear-positive PTB to smear-negative PTB. The discrepancies between notifications and estimations in both TB morbidity and mortality of children need to be addressed urgently.

## Background

Tuberculosis (TB), an infectious disease caused by bacteria and spread through air, is preventable and treatable. However, the World Health Organization (WHO) estimates that 10.0 million people developed TB disease and 1.6 million died globally, making TB the leading cause of death (above HIV/AIDS) from a single infectious agent in 2017 [1]. One million patients who suffered from TB are < 15 years of age, with 0.2 million death each year, corresponding to 23 children die of TB per hour [1].

TB in children has attracted increasing attention since the first publication of Guidance for National Tuberculosis Programmes on the Management of Tuberculosis in Children in 2006 [2]. However, the global TB burden on children was first reported till 2012 [3]. Still more than half (55%) of these patients were missed (under-reporting or under-diagnosis) in 2017 [1]. The exact epidemiological characteristics of childhood TB are unknown due to insufficient public health concern (deemed as less infection, not a major source of disease transmission) [4], the wide spectrum of disease (pulmonary and extra-pulmonary) [5], and the diagnostic difficulties (non-specific symptoms, hard to obtain specimens, the paucibacillary nature, non-specific tests, low sensitivity of those tests, .etc) [4–8].

Up to 26% of the global population and 43% of the population in low-income countries were children [9]. Child with TB is at high risk for severe disease and death [10, 11]. Even the one with a latent infection or who has gotten treatment success (cure or completion) could become a reservoir of TB reactivation or relapse [12]. Moreover, child with TB represents recent transmission and can be regarded as a sentinel event for ongoing transmission within community [12–14]. Thus, childhood TB plays a vital role in global TB control. To eradicate TB, the End TB Strategy outlines an overall target of reducing global TB incidence and mortality by 90 and 95% respectively by the year of 2035 [15]. Failing to monitor the incidence and mortality of childhood TB makes it hard to estimate whether these targets will be met.

This study aims to describe characteristics and epidemiology of PTB among patients aged < 18 years in seven cities of Shandong Province, China, from 2005 (the first time that free treatment was available for smear-negative PTB) to 2017.

## Methods

### Study population and data collection

TB cases in this study were collected at the city level from the Tuberculosis Information Management System, Chinese Center for Disease Control and Prevention (CDC). TB has to be reported and registered in the CDC system within 24 h and the failure to report will be a crime in China. This study used convenience sampling method and included seven cities (Dezhou, Jinan, Jining, Liaochen, Linyi, Weifang, and Yantai) in Shandong province. Cities selection were based on geographic location and it was intended to reflect the range of TB burdens and clinical capacities. This study covered 54% population, 50% health institutions, and 51% sanitation stations of Shandong province. For analysis, we included all PTB cases aged < 18 years during 2005–2017. Data on demographic, clinical information, disease verification, and treatment outcome were collected. Shandong Statistical Yearbook provided population data annually.

### Laboratory methods and laboratory quality control

All patients with probably PTB (cough or fever for > 2 weeks, weight loss or failure to thrive, TB contact history, abnormal chest radiograph) were requested to submit three pulmonary samples for acid-fast bacilli (AFB) with Ziehl-Neelsen smear microscopy before the initiation of treatment. Pulmonary samples were collected by expectoration, gastric aspiration, sputum induction, and bronchoscopy. The Katharin Hsu Center has been responsible for laboratory quality assurance in Shandong Province since 2004.

### Data inclusion and definitions

At least two sputum smear examinations positive for AFB or one sputum smear examination positive for AFB plus abnormal chest radiograph consistent with active PTB were essential to the diagnose of smear-positive PTB [2]. The diagnose of smear-negative PTB was made according to the combination of clinical symptoms with ineffective broad-spectrum antibiotics treatment, effective anti-TB treatment, radiological abnormalities consistent with active PTB, and close contact with TB patients, .etc. [2]. All PTB cases aged < 18 years without HIV co-infection were included in this study.

The one who shared an enclosed space with TB patients (usually family members or classmates) in the past 3 months was defined as having a close contact history [16]. In China, all the classmates study in the same classroom every academic year before entering university. The floating population, or *liudong renkou*, referred to the one who resided in a county/district other than his or her local registered residence (*hukou*) [17]. We only included patients aged < 18 years. The majority of “floating population” should be students who went school in another county/district in this study. Treatment outcomes were defined according to the *Definitions and reporting framework for tuberculosis-2013 revision* [18]. For analysis purposes, “cured” and “treatment completed” were classified as “treatment success”, whereas “treatment failed”, “died”, “lost to follow-up”, and “not evaluated” were classified as “poor treatment outcome”. The one who had never taken or had taken anti-TB drugs for < 1 month was defined as a new case. Among patients with treatment success, the one who was diagnosed with a recurrent episode of TB, whether a true relapse or a new reinfection, was defined as a relapse case [18].

### Statistical analysis

Continuous variables were summarized with mean and standard deviation (SD); categorical variables were summarized as proportions. We compared characteristics between smear-positive PTB and smear-negative PTB using logistic regression analysis. Univariable logistic regression models were performed to derive crude odds ratios (ORs). In multivariable logistic regression model, demographic and clinical variables were included to derive adjusted ORs and 95% confidence intervals (CIs).

The incidence (per 100,000) was calculated as annual PTB cases divided by annual population. The overall incidence, smear-positive/smear-negative PTB incidence, new/relapse PTB incidence, monthly incidence and city-specific incidence were calculated. We used joinpoint regression models [19] to examine incidence trends from 2005 to 2017. Annual percentage changes (APCs) were used to describe trends. We used the Z test to assess whether an APC was significantly different from zero. The term of increase or decrease was used to describe the trend when a significant positive or negative slope (APC) was observed. A non-significant (*P* ≥ 0.05) APC was described as stable and indicated that the incidence was maintained at a relatively stable level.

All analyses were performed using SAS 9.2 software (SAS Institude) and Joinpoint (version 4.3.1). A *P* < 0.05 was considered statistically significant.

## Results

During 2005–2017, 260,726 PTB cases were reported in seven cities of Shandong. A total of 6283 (2.4%) cases aged < 18 years were included, the mean age was 15.6 years.

**Fig. 1:**
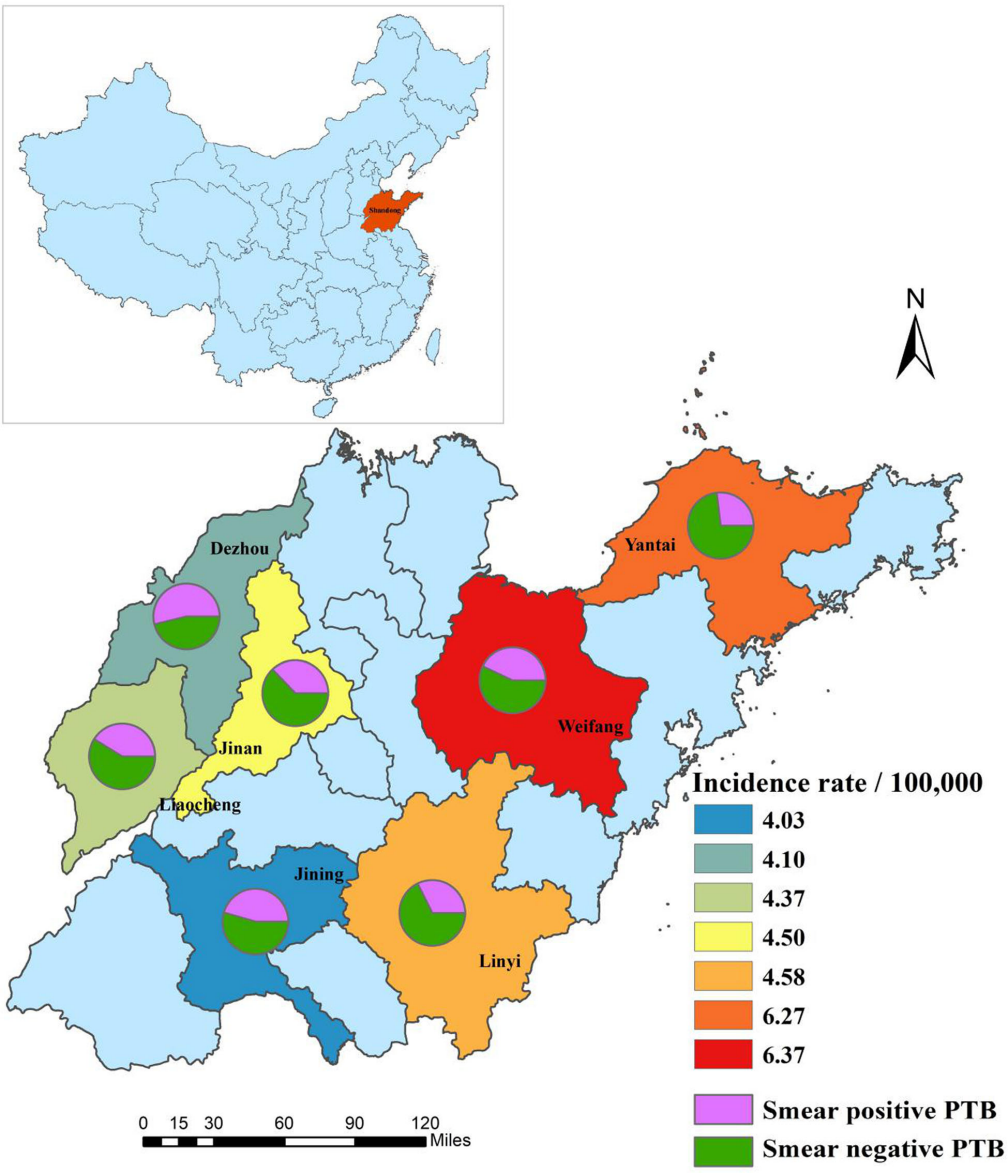
Averaged incidence of childhood pulmonary tuberculosis in seven cities of Shandong, China, 2005–2017

Of the 6283 children with PTB, 5234 (83.3%) were > 15 years of age, 834 (13.3%) were > 10 but < 15 years of age, 139 (2.2%) were > 5 but < 10 years of age, and only 76 (1.2%) were < 5 years of age. Males accounted for 56.5%, Han 98.9%, and floating population 9.2%. The smear-positive PTB were verified in 2472 (39.3%) cases, whereas 3811 (60.7%) were smear-negative PTB. Up to 6194 (98.6%) PTB were new cases and only 89 (1.4%) were relapse cases. At least 15% patients had a TB contact history. Linyi and Weifang had the heaviest disease burden, accounting for 43% of all childhood PTB (Table 1). Figure 1 demonstrates the disparity of childhood PTB in seven cities of Shandong, China, 2005–2017.

**Table 1:** Characteristics of 6283 PTB patients aged < 18 years in seven cities of Shandong, 2005–2017

Variables	Overall *N* = 6283	%
Sex		
Male	3553	56.5
Female	2730	43.5
Age (years)		
0–4	76	1.2
5–9	139	2.2
10–14	834	13.3
15–17	5234	83.3
Ethnic group		
Han	6215	98.9
Other	68	1.1
Treatment history		
New case	6194	98.6
Relapse case	89	1.4
Disease verification		
Smear-positive PTB	2472	39.3
Smear-negative PTB	3811	60.7
TB contract	958	15.2
Population		
Local population	5705	90.8
Floating population	578	9.2
Geographical location		
Dezhou	614	9.8
Jinan	638	10.2
Jining	864	13.8
Liaocheng	711	11.3
Linyi	1370	21.8
Weifang	1326	21.1
Yantai	760	12.1
Treatment outcomes		
Cured	2342	37.3
Completed	3575	56.9
Died	10	0.2
Failed	34	0.5
Transferred out	11	0.2
Others	311	4.9

*PTB* pulmonary tuberculosis

Among 5917 (94.2%) of the cases with treatment success, 2342 (37.3%) children were cured and 3575 (56.9%) were completed. Whereas among 366 (5.8%) cases with poor treatment outcomes, 10 (0.2%) children were died, 34 (0.5%) were failed, 11 (0.2%) were transferred out, and 311 (4.9%) were not evaluated (Table 1).

Table 2 illustrates the characteristics of smear-positive PTB and smear-negative PTB among children in detail. Smear-positive PTB were more likely to relapse (ORa 10.9, 95% CI 5.7, 20.6) and aged 15 to17 years (ORa 1.7, 95% CI 1.0, 2.8) than smear-negative PTB. Smear-negative PTB were more likely to be male (ORa 0.8, 95% CI 0.7, 0.8) and to be floating population (ORa 0.3, 95% CI 0.2, 0.3) than smear-positive PTB. There was no statistically significant difference in the treatment outcomes between smear-positive PTB and smear-negative PTB. Additional comparison of treatment outcomes (treatment success, poor treatment outcome) between local population and floating population showed no statistically significant difference (*P* = 0.8) either.

**Table 2:** Characteristic of smear-positive PTB and smear-negative PTB in patients aged < 18 years in seven cities of Shandong, 2005–2017

	Disease verification		ORc	ORa	95%CI
	Smear-positive (*n* = 2472) No. (%)	Smear-negative (*n* = 3811) No. (%)			
Sex					
Male	1297 (52.5)	2256 (59.2)	0.760	0.765	0.689–0.849
Female	1175 (47.5)	1555 (40.8)	Reference	Reference	
Age (years)					
0–4	23 (0.9)	53 (1.4)	Reference	Reference	
5–9	33 (1.3)	106 (2.8)	0.717	0.773	0.406–1.472
10–14	258 (10.4)	576 (15.1)	1.032	1.074	0.632–1.825
15–17	2158 (87.3)	3076 (80.7)	1.617	1.678	1.006–2.798
Ethnic group					
Han	2455 (99.3)	3760 (98.7)	1.957	0.767	0.420–1.401
Other	17 (0.7)	51 (1.3)	Reference	Reference	
Population					
Local population	2376 (96.1)	3329 (87.4)	Reference	Reference	
Floating population	96 (3.9)	482 (12.7)	0.279	0.276	0.218–0.349
Treatment history					
New case	2394 (96.8)	3800 (99.7)	Reference	Reference	
Relapse case	78 (3.2)	11 (0.3)	11.248	10.860	5.713–20.643
Treatment outcome					
Cured and completed	2327 (94.1)	3590 (94.2)	Reference	Reference	
Died	6 (0.2)	4 (0.1)	2.314	3.376	0.834–13.665
Failed	18 (0.7)	16 (0.4)	1.736	1.403	0.694–2.834
Others	121 (4.9)	201 (5.3)	0.929	0.942	0.742–1.196

*CI* confidence interval, *ORa* adjusted odds ratio, *ORc* crude odds ratio, *PTB* pulmonary tuberculosis

**Fig. 2:**
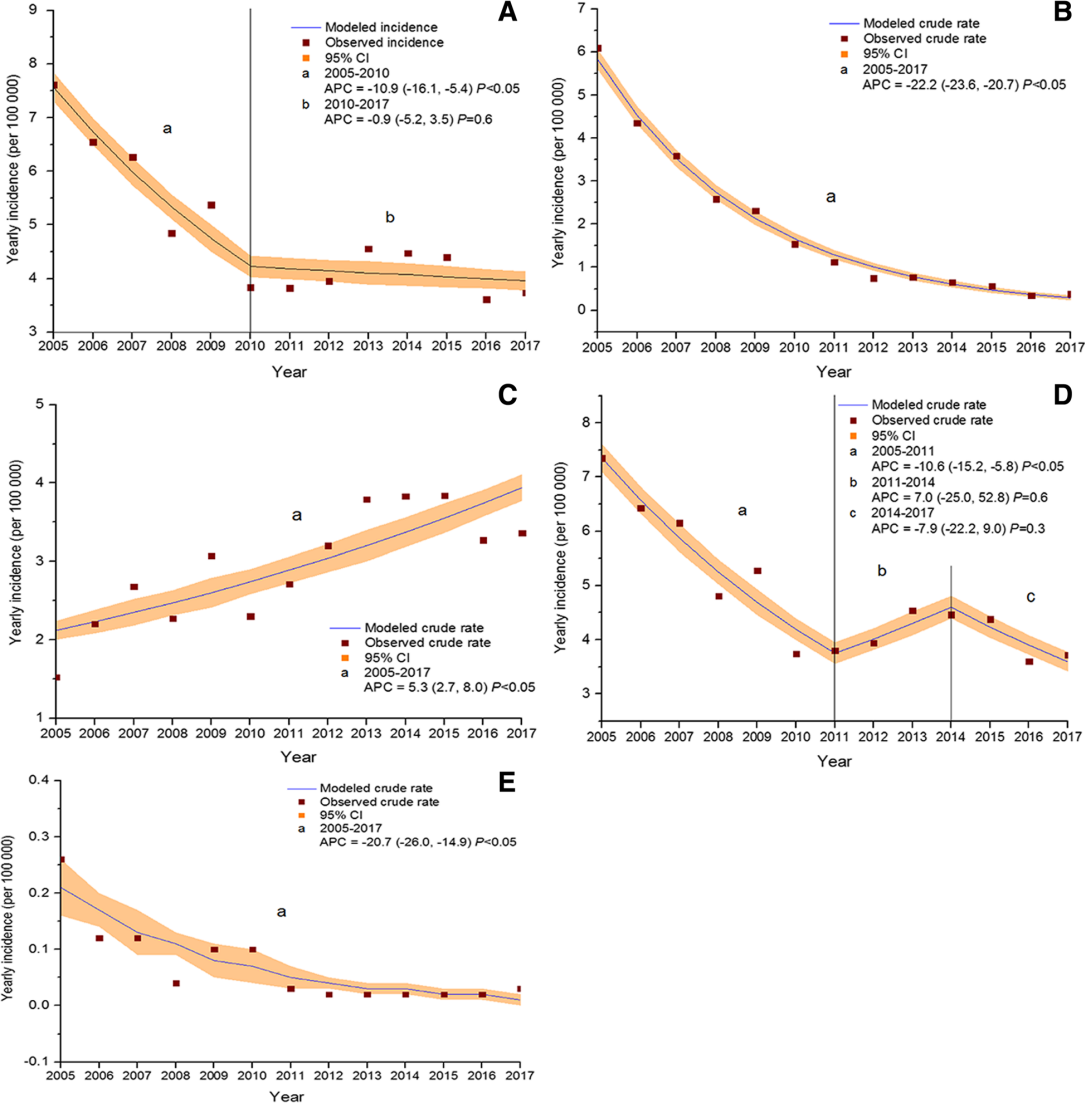
Joinpoint regression showing trends in incidence of pulmonary tuberculosis cases aged < 18 years. **a** trends in incidence of overall PTB cases; **b**) trends in incidence of smear-positive PTB cases; **c**) trends in incidence of smear-negative PTB cases; **d**) trends in incidence of new cases; **e**) trends in incidence of relapse cases; APC: annual percentage change, PTB: pulmonary tuberculosis

The incidence of overall PTB among children declined from 7.62 to 3.74 per 100,000 population during the study period. The joinpoint regression indicated an APC of − 10.9% (95% CI -16.1, − 5.4; *P* < 0.05) from 2005 to 2010 and then it remained largely stable (Fig. 2a). The incidence of smear-positive PTB among children declined from 6.09 to 0.38 per 100,000 population during 2005–2017. The joinpoint regression indicated an APC of − 22.2% (95% CI -23.6, − 20.7; *P* < 0.05) (Fig. 2b). The incidence of smear-negative PTB among children increase from 1.52 to 3.36 per 100,000 population during 2005–2017. The joinpoint regression indicated APC of 5.3% (95% CI 2.7, 8.0; *P* < 0.05) (Fig. 2c). The incidence of new cases among children declined from 7.35 to 3.72 per 100,000 population during the study period. The joinpoint regression indicated an APC of − 10.6% (95% CI -15.2, − 5.8; *P* < 0.05) from 2005 to 2011 and then it remained largely stable (Fig. 2d). The incidence of relapse cases among children declined from 0.26 to 0.03 per 100,000 population during 2005–2017. The joinpoint regression indicated an APC of − 20.7% (95% CI -26.0, − 14.9; *P* < 0.05) (Fig. 2e).

**Fig. 3:**
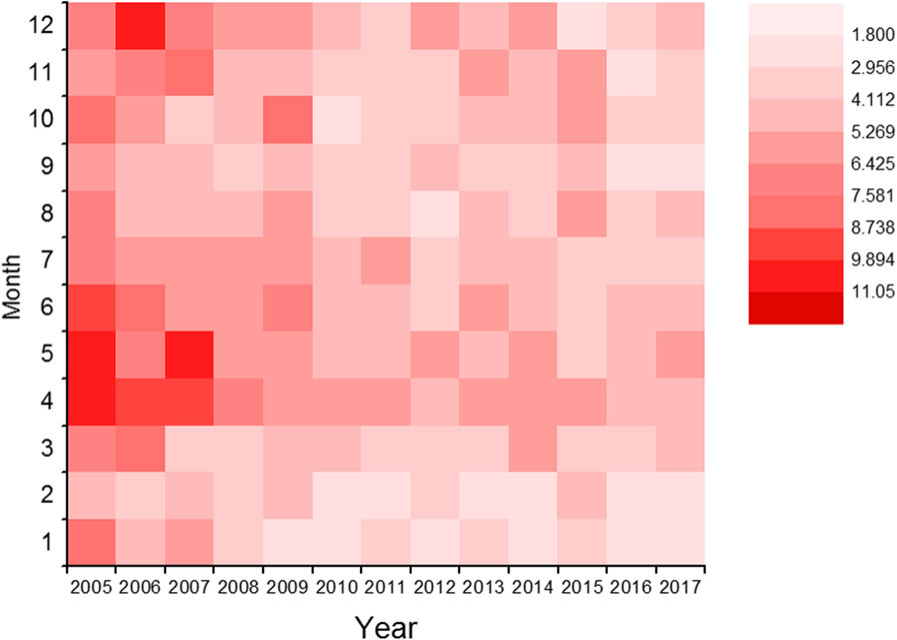
Trends in incidence stratified by reported month among pulmonary tuberculosis cases aged < 18 years

The highest incidence grouping by reported months occurred during April to June 2005. Ten of the 12 months showed significantly decreasing trends in incidence during 2005–2017: January, March, April, May, June, July, September, October, November, and December (*P* < 0.05). The joinpoint regression indicated an APC of − 21.6% for January and − 13.5% for April (*P* < 0.05) from 2005 to 2009 and then it remained largely stable. Moreover, the incidence remained largely stable in February and August during 2005–2017 (*P* = 0.10 and *P* = 0.20 respectively) (Table 3, Fig. 3).

**Table 3:** Annual percentage change in incidence stratified by reported month among PTB cases aged < 18 years in seven cities of Shandong, 2005–2017

Month	Year	Trend	APC (95%CI)	*P* value
Jan	2005–2009	Decrease	−21.6% (− 34.8, − 5.7)	< 0.05
	2009–2017	Stable	−0.0% (−8.0, 8.7)	1.0
Feb	2005–2017	Stable	−3.8% (−7.7, 0.3)	0.1
Mar	2005–2017	Decrease	−4.4% (−8.1, −0.6)	< 0.05
Apr	2005–2009	Decrease	−13.5% (−21.2, − 5.0)	< 0.05
	2009–2017	Stable	−2.7% (−6.6, 1.4)	0.2
May	2005–2017	Decrease	−5.8% (−9.2, −2.3)	< 0.05
Jun	2005–2017	Decrease	−5.8% (−8.6, −2.9)	< 0.05
Jul	2005–2017	Decrease	−5.1% (−6.5, −3.7)	< 0.05
Aug	2005–2017	Stable	−2.5% (−6.0, 1.2)	0.2
Sep	2005–2017	Decrease	−4.3% (−7.4, −1.1)	< 0.05
Oct	2005–2017	Decrease	−5.7% (−10.4, −0.8)	< 0.05
Nov	2005–2017	Decrease	−4.9% (−9.2, − 0.3)	< 0.05
Dec	2005–2017	Decrease	−5.9% (−9.7, −1.9)	< 0.05

*APC* annual percentage change, *PTB* pulmonary tuberculosis

## Discussion

To our knowledge, previous national TB epidemiological surveys in China either only covered patients aged ≥15 years or dealt with patients of all ages as a whole [20]. The exact epidemiological characteristic of childhood PTB is limited in China. This retrospective cohort review provides original, large population and long-term based data on the epidemiological and clinical characteristics of PTB among children (aged < 18 years) in the second largest province located in the eastern coast of China. It shows that: 1) childhood PTB accounted for 2.4% of all PTB cases; 2) the overall incidence of childhood PTB declined during 2005–2010, then it remained stable in the following 7-year; 3) the disease burden had shifted from smear-positive PTB (6.09 to 0.38 per 100,000 population) to smear-negative PTB (1.52 to 3.36 per 100,000 population) during the study period; and 4) the overall treatment success occurred among 94% childhood PTB and 10 (0.2%) children died in this study.

The reported childhood PTB in this study represented only the tip of the iceberg. China had the second largest amount of TB, accounting for 9% of global TB burden [1]. A predicted proportion of more than 5% TB cases in China were children < 15 years of age in 2010 [21]. Moreover, 52% of childhood TB patients were young children who aged < 5 years according to the WHO report [22]. In this study, only 0.4% of PTB patients were aged < 15 years and young children accounted for 0.03% of all PTB patients. Due to difficulties in sample collection, the paucibacillary nature, non-specific tests, and the low sensitivity of AFB smear and culture (< 5 and 15% respectively), children especially young children were less likely to be bacteriological confirmed as TB [6–8]. A clinical diagnose of TB can also be impeded among children, because they always had non-specific symptom [5] and were easily misdiagnosed as another disease (for example, pneumonia) [23, 24]. Globally, up to 69% of childhood PTB who aged < 5 years and 40% of childhood PTB who aged < 15 years were missed [22]. The gap between estimations and notifications should therefore spur not hopelessness, but action.

The overall incidence of childhood PTB declined significantly with the disease burden shifted from smear-positive PTB to smear-negative PTB during the study period. The trends in incidence of overall PTB (decreased), smear-positive PTB (decreased), and smear-negative PTB (increased) among children in this study were similarly with that among patients of all ages in other studies [20, 25]. The significant decline of smear-positive PTB mainly attributed to the high-quality directly observed treatment, short-course (DOTS) strategy in China [20]. Due to the lack of bacteriological indicators, the diagnose of smear-negative PTB was complicated and many factors could influence the identification of these patients [25]. China was one of the low-income countries, where sophisticated and expensive modern technologies (TB antibody test, adenosine deaminase, interferon-γ release test, and tuberculosis-infected T-cell detection, high-resolution computed tomography) developed slowly and continuously [26]. The probably reason for the increased incidence of smear-negative PTB in this study was the gradually development and utilization of modern diagnostic methods, and the gradually increased public concern on TB. More investments to ensure these existed methods fully implemented, development of new accurate and prompt diagnostic methods toward suspected children are vital.

The overall treatment success occurred among 94% childhood PTB and 10 (0.16%) children died in this study. Childhood TB patients who were promptly diagnosed and treated tend to do well (< 1% mortality) [27]. About 95% patients were actively followed-up in this study, thus it may contribute to the high “treatment success” rates. However, among childhood TB (including patients both diagnosed and unrecognized), an estimated 96% of deaths occurred in patients who did not access TB management and 80% in patients < 5 years which were less likely to be diagnosed, but more likely to suffer severe forms of TB [5]. TB is a preventable and curable disease but we need to identify these undetected patients in the first place. Although it is inappropriate for children, the current TB case-finding strategy mainly relies on passive case-finding, waiting for the symptomatic individuals voluntarily seeking medical care and treatment in China. Moreover, about 75% (of 1.3 million eligible household contacts < 5 years) patients did not access preventive therapy worldwide [22]. If the case-detection ratio for children were improved or children with high risk were all given preventive therapy, then many lives will be saved.

Medical factors, income, education, geography, environment, customs, .etc. were associated with TB prevalence [28, 29]. Time and regional inequity of childhood PTB burden were also demonstrated in this study. Locally comprehensive strategies and methods for TB control and prevention should be formulated.

Childhood TB is an important indicator for recent transmission and provides the reservoir for the disease [12–14]. Without the successful detection and treatment of TB infection or disease in children, the target of achieving zero deaths for childhood TB by 2025 will be difficult to meet [30]; control of TB still faces huge challenges.

This study had some limitations. Firstly, as a retrospective study, few information of the included patients (nutritional condition, living conditions, education) and their source cases (origin of infection) were provided by the medical records. Second, this study only included children with PTB, because reporting of pleural TB and extra-pulmonary TB was not mandatory. Third, only one province on the eastern coast of China was examined, the economic, ethnicity, and regional disparities limited the generalizability of the results.

## Conclusion

The childhood PTB cases reported in this study only represent a small fraction of the total TB burden. The discrepancies between notifications and estimations in both TB morbidity and mortality of children need to be addressed urgently. Disease burden has shifted from smear-positive PTB to smear-negative PTB in children. More attention to ensure the existing methods fully implemented and the development of new accurate and prompt diagnostic methods are important for future TB control.

## Abbreviations

AFB: Acid-fast bacilli
APC: Annual percentage change
CDC: Center for Disease Control and Prevention
CI: Confidence interval
OR: Odds ratio
PTB: Pulmonary tuberculosis
TB: Tuberculosis
WHO: World Health Organization
